# Studying Depression, Anxiety, Distress and Somatisation in a Community Sample of 2,425 Adults in Greece

**DOI:** 10.14806/ej.26.1.972

**Published:** 2021-10-08

**Authors:** Maya Louvardi, Panagiotis Pelekasis, Flora Bacopoulou, Dimitrios Vlachakis, George P. Chrousos, Christina Darviri

**Affiliations:** 1 Postgraduate Course of Science of Stress and Health Promotion, School of Medicine, National and Kapodistrian University of Athens, Athens, Greece; 2 University Research Institute of Maternal and Child Health & Precision Medicine and UNESCO Chair on Adolescent Health Care, National and Kapodistrian University of Athens, Aghia Sophia Children’s Hospital, Athens, Greece; 3 Laboratory of Genetics, Department of Biotechnology, School of Applied Biology and Biotechnology, Agricultural University of Athens, Athens, Greece; 4 Lab of Molecular Endocrinology, Center of Clinical, Experimental Surgery and Translational Research, Biomedical Research Foundation of the Academy of Athens, Athens, Greece

## Abstract

A growing part of the literature has focused on depression, anxiety, distress and somatisation. Identifying their prevalence and populations at risk is essential to form relevant interventions. The aim of this study was to examine the prevalence and associated factors of distress, depression, anxiety, and somatisation in a community adult sample in Greece. Participants were recruited from two Greek cities; Giannitsa in the northern area and Athens in the southern area of the country, and completed sociodemographic assessments, as well as the 4-Dimensional Symptom Questionnaire (4-DSQ), a self-reported instrument assessing depression, anxiety, distress, and somatisation.

A total of 2,425 adults, females (60.1%) and males (39.9%), 18 to 84 years of age (mean age±SD, 46.98±9.57 years) participated in the study. Mental health symptoms were reported by 10.8% for depression, 12% for anxiety, 13% for distress and 5.3% for somatisation. Females scored higher than males in anxiety, distress, and somatisation (p=0.000 in all cases), while there were no significant sex differences in depression (p=0.593). Statistically significant associations were found between age and depression, anxiety and distress (p=0.000 in all cases), since those between 18–34 years of age had higher scores than the older age groups in all variables. Higher scores of depression, anxiety and distress were reported by students and unemployed participants (p=0.000 in all cases) than participants with other occupations.

This study mapped several sociodemographic groups with worse mental health. Studies in representative population samples are needed to guide public health interventions to improve the well-being of high-risk populations.

## Introduction

According to the Global Burden of Disease Study, the prevalence of mental health disorders, such as depressive and anxiety disorders, has increased during the previous decades (Global Burden of Disease Study 2013 Collaborators, 2015). A wide range of changes in the way of living, which were intensified after the middle of the 20th century, such as urbanization, consumerism and secularization, are held responsible for the increased prevalence of these disorders (Hidaka, 2012).

Apart from clinically significant disorders, a major part of the research has focused on the impact of stress, which is causally related to the onset of several psychiatric disorders (Hammen, 2005; Pittenger and Duman, 2008). In addition, stress is related to various somatic disorders, such as coronary heart disease, breast cancer, multiple sclerosis, and diabetes type II (Antonova *et al.*, 2011; Kelly and Ismail, 2015; Lin *et al.*, 2013; Lloyd *et al.*, 2005; McKay *et al.*, 2017; Wirtz and von Känel, 2017). Hence, stress should be considered as a major threat for public health.

Due to the aforementioned aggravating effects, it is of most importance to form a mechanism explaining the pathway from stress to chronic morbidity. As supported by several prominent professors in stress research, this effect can be explained by the negative impact of stress on the cardiovascular, gastrointestinal, muscular, immune and pulmonary function (Chrousos and Gold, 1992; McEwen, 1998). Somatisation refers to the process in which stress is experienced at a somatic level, affecting the homeostasis of such systems (Dantzer, 1995). As supported by Ford (1997), impaired capacity to communicate psychological experiences related to stress leads to somatisation.

Despite the fact that stress is experienced by literally everyone, some people are predisposed for increased stress levels, based on their sociodemographic profile. For example, the unemployed are a group experiencing high stress levels (Frasquilho *et al.*, 2016). Yet, the impact of such factors is influenced by cultural parameters, highlighting the need to investigate such effects on different contexts (Marsh and Alvaro, 1990). Indeed, the heterogeneity of cultural norms indicates the necessity to study the mental health phenomena in divergent cultural contexts, to investigate if the recorded effects are common across the different contexts or not (Robson, 2002).

Based on the aforementioned evidence, it is of most importance to map high-risk populations for stress, anxiety, depression and somatisation, to provide a target for public health policies. The aim of this study was to examine the prevalence and associated factors of distress, depression, anxiety and somatisation in a community adult sample in Greece.

## Materials, Methodologies and Techniques

### Study design

The design of the study was cross-sectional. Recruitment to the study was carried out in two different cities, in Athens, the capital city of Greece (664,046 inhabitants) and in Giannitsa, a city in northern Greece (29,789 inhabitants). The recruitment process began on 6 December 2018 and ended on 16 May 2019.

### Participants

Study participants were adults, able to communicate verbally and in writing in Greek.

### Measurements

#### Sociodemographic data:

Participants’ sociodemographic data included age (years), sex (male / female), family status (unmarried living alone/ unmarried living with a partner/ married/ widowed/ divorced), number of children, educational level (Primary / Gymnasium / Lyceum / Tertiary / MSc / PhD), smoking status (current smoker / occasional smoker / non-smoker), and occupational status (unemployed / student / private sector worker / public sector worker / freelancer or businessman-woman / pensioner / house worker).

#### The 4-Dimensional Symptom Questionnaire:

The 4-Dimensional Symptom Questionnaire (4-DSQ) is a self-reported instrument, including 50 items scored on a five Likert-type scale (zero=no to four=very much or always). This instrument includes four different subscales, measuring distress, depression, anxiety and somatisation (Terluin *et al.*, 2006). The 4-DSQ has been validated in Greek (Tsourela *et al.*, 2013). The range of α level was 0.90 for depression, 0.89 for anxiety, 0.92 for distress and 0.87 for somatisation.

### Procedures

Prior to the beginning of the study, approval was obtained by the ethics committee of the Medical School of the National and Kapodistrian University of Athens. The study was in line with the Declaration of Helsinki. The recruitment process was carried out by a health visitor (ML) in Universities, public services, private companies and public spaces. A total of 3,000 participants were invited to participate and were informed about the purpose of the study, in face-to-face meetings. Those agreeing to participate provided informed consent and completed the assessments instantly or returned them on another day, based on relevant communication. The data collection was anonymous and confidential. The assessments were returned by 2,555 participants (response rate 85.17%) and 2,425 completed questionnaires were further analysed. The average response time was approximately ten minutes.

### Statistical analysis

The statistical analysis was carried out with the SPSS vol. 25 statistical software for Windows (Chicago Inc.). At first, descriptive statistics were applied to calculate the sociodemographic characteristics of the study sample. Descriptive statistics were also used to calculate the prevalence of elevated depression, anxiety, distress and somatisation levels, based on the cut-offs suggested by the developers of the 4-DSQ instrument (Terluin *et al.*, 2006). Subsequently, inductive statistics were applied to search for relationships between the sociodemographic data of the study and the participants’ score on the 4-DSQ. The independent samples T-Test was used when the sociodemographic variables were binary, and ANOVA when the sociodemographic variables had more than two values. Bonferroni post-hoc test and Mean Difference (M.D.) calculation followed the ANOVA analysis, in case of statistical significance. The level of significance was set at 0.05 for all the analyses.

## Results

The descriptive data of the study sample are presented in Table 1.

Most participants were females (60.1%), between 18–34 years of age (52.3%), living in Athens (73.9%), not having children (65.3%). Concerning family status, the majority were unmarried, living alone (42.1%), many were married (34.4%), fewer were unmarried living with a partner (17.2%), while the minority were widowed or divorced (6.2%). As for the educational status, 42.6% were of tertiary education, while 27.3% were lyceum graduates. Regarding their occupational status, 33% were private sector workers, 23.1% freelancers, 19.5% public sector workers and 16.3% were students. Finally, 57.2% were non-smokers, 30.6% were smokers and 12.2% were occasional smokers.

The prevalence of depression, anxiety, distress and somatisation in the study sample is presented at Table 2. Most participants had normal or mild levels of depression (74.4%), anxiety (65.3%), distress (57.1%) and somatisation (70.8%).

The prevalence of severely elevated levels of depression, anxiety, distress and somatisation by participants’ sex is presented in Table 3. Females had higher scores than males in all sub-scales of 4-DSQ.

As indicated in Table 4, statistically significant associations were found between family status, educational status and occupational status in all sub-scales of the 4-DSQ. Concerning the age and number of the children, statistically significant associations were found in all sub-scales except for the somatisation subscale. Concerning participants’ sex and smoking status, statistically significant associations were found in all sub-scales except the depression subscale. More specifically, males had lower scores than females (p=0.000). As for the area of residence, no association was found with the depression, anxiety, distress or somatisation levels.

Post-hoc analysis demonstrated several differences between groups. Specifically, participants aged 18–34 years showed higher levels of distress (M.D. 1.86, p=0.000), anxiety (M.D. 1.04, p=0.000) and depression (M.D. 0.37, p=0.028) than the 35–49 age group. Also, participants aged 18–34 years reported higher levels of distress (M.D. 2.72, p=0.000) and anxiety (M.D. 1.40, p=0.000) than the 50–64 age group.

With regards to family status, statistically significant differences were found between married and unmarried living with a partner participants; the latter group reported higher levels of distress (M.D. −2.50, p=0.000), anxiety (M.D. −1.00, p=0.001), depression (M.D. −0.488, p=0.025), and somatisation (M.D. 1.072, p=0.019). Nevertheless, married participants demonstrated lower levels of distress (M.D. −2.37, p=0.000), anxiety (M.D. 1.25, p=0.000), depression (M.D. −0.517, p=0.001), and somatisation (M.D. 1.003, p=0.027) than those living alone.

With respect to children, participants who did not have children reported higher scores in distress (M.D. 1.92, p=0.000), anxiety (M.D. 0.80, p=0.008), and depression (M.D. 0.498, p=0.013) than those with one child, as well as higher levels of distress (M.D. 2.43, p=0.000), and anxiety (M.D. 1.15, p=0.000) than those with two children.

With reference to occupational status, the unemployed participants reported higher levels of distress (M.D. 3.55, p=0.001), anxiety (M.D. 1.99, p=0.000), and depression (M.D. 1.17, p=0.002) than the public sector workers, as well as higher levels of distress (M.D. 2.88, p=0.002), anxiety (M.D. 2.01, p=0.000), and depression (M.D. 1.02, p=0.012) than the freelancers. Furthermore, the unemployed participants reported higher levels of distress (M.D. 2.40, p=0.015), anxiety (M.D. 1.39, p=0.034), and depression (M.D. 0.877, p=0.045) than the private sector workers. Also, students reported higher levels of distress (M.D. 3.35, p=0.000), and depression (M.D. 0.776, p=0.005) than public sector workers, higher levels of distress (M.D. 2.19, p=0.001) than private sector workers, and higher levels of distress (M.D. 2.68, p=0.000), anxiety (M.D. 1.94, p=0.000), and depression (M.D. 0.621, p=0.048) than the freelancers. Moreover, private sector workers reported higher levels of somatisation than public sector workers (M.D. 1.24, p=0.049) and freelancers (M.D. 1.51, p=0.002).

Likewise, statistically significant differences were found between smokers and non-smokers with the latter group reporting lower scores in distress (M.D. 1.13, p=0.006) and somatisation (M.D. 1.28, p=0.000), as well as between occasional smokers and non-smokers, with the latter group reporting lower scores in distress (M.D. 1.33, p=0.027).

With regards to the educational status, PhD students reported lower levels of distress (M.D. −3.966, p=0.046) than the Lyceum graduates. As for anxiety, statistically significant differences were noted between Lyceum and MSc participants, (M.D. 1.094, p=0.002), as well as between Lyceum and tertiary education participants (M.D. 0.663, p=0.028). As for depression, statistically significant differences were found between Lyceum and tertiary educational level (M.D. 0.490, p=0.033) participants, Lyceum and MSc participants (M.D. 0.584, p=0.020), Lyceum and vocational training (M.D. 0.632, p=0.014) participants. Differences were also noted between PhD participants and other educational groups; more specifically PhD participants, demonstrated lower distress levels (M.D. −1.908, p=0.034) than Gymnasium participants, as well as lower levels of somatisation than tertiary education participants (M.D. −3.375, p=0.013), Lyceum graduates (M.D. −3.547, p=0.008), vocational training graduates (M.D. −4.331, p=0.001) and Gymnasium graduates (M.D. −4.263, p=0.042).

## Discussion

This study investigated the prevalence of depression, anxiety, distress and somatisation in an adult community sample in Greece, as well as associated factors. In the sample studied, 10.8% of the participants had severe depressive symptoms, while anxiety, distress and somatisation were reported by 12%, 13% and 5.3% of the participants, respectively. Higher prevalence was noted for moderate distress (43%) compared to the other studied parameters (24.8% −34.7%). As for the associated factors, there were significant sex differences in anxiety, distress, and somatisation, since females had higher scores, while there were no significant differences for depression. Concerning age, higher scores of depression, anxiety, distress and somatisation were found for younger participants, especially for those aged 18–34 years old. Higher scores of depression, anxiety and distress were found for students and the unemployed, whereas private sector workers had higher scores of somatisation compared to public sector workers and freelancers. Also, participants who were married and those with children had lower scores of distress, anxiety, depression and somatisation, while the unmarried living with a partner or not and those with no children had higher scores in all subscales. Smokers experienced worse mental health and somatisation compared to the others. Finally, participants with a PhD had lower scores in all subscales. At last, there are no statistically significant differences in the parameters studied between participants residing in the two cities in the northern and southern areas of the country.

The prevalence of severe depressive symptoms (10.8%) in this study is similar to that reported in other countries. For example, Johansson *et al.* (2013) found that 10.8% of the Swedish general population had clinically significant depressive symptoms, while Doğan *et al.* (2011) found that 12.8% of the general population in Turkey had such symptoms. In addition, Johansson *et al.* (2013) found that 14.7% of the responders had severe anxiety, which is slightly higher compared to the 12% found in the present study. As for somatisation, the reported 5.3% of severe symptoms in the present study is quite similar to the 5% found by Lee *et al.* (2015), who investigated somatisation in the general population in Hong Kong and to the 6.3% of somatoform disorders found by Wittchen *et al.* (2011) across the EU countries. Thus, it seems that the prevalence of depression, anxiety and somatisation is quite similar across different countries and is not strongly affected by cultural norms.

Most of the study findings concerning the associated factors are in line with previous research. For example, the findings of the present study support that those living in a family and having children have better mental health and lower somatisation compared to the others. This finding confirms the already known theories about the protective effect of social ties on human health (Moore and Kawachi, 2017). However, a finding that is not in line with other studies is the absence of statistically significant differences between males and females in depression, since according to the World Health Organization (2017) males experience lower depression levels.

Another finding that is in line with the previous literature concerns the association of smoking status with mental health and somatisation. As supported by West (2017), this association is quite common in the literature, although there is no commonly accepted mechanism of why smokers experience worse mental health. According to his point of view, this effect could be explained by the higher levels of life satisfaction of the non-smokers. An alternative explanation could be that smokers are aware of the potentially harmful effect of smoking, as well as that they feel guilt for placing their health under threat. These findings are in line with research in patients affected by smoking-related diseases, especially lung cancer (Weiss *et al.*, 2017). In addition, it could be supported that smokers have higher trait anxiety levels, since smoking is considered as a maladaptive way to set anxiety under control (Wiggert *et al.*, 2016).

As for the effect of educational status on the components of mental and physical well-being, the findings of the present study contradict previous research supporting that high education in general leads to better mental and physical health outcomes (Berkman *et al.*, 2014), since only those with extremely high educational level were found to be protected. It could be supported that in the Greek market there is no strong association between education and work positions, since many people do not work on their field of expertise, a problematic condition present in Greece even before the economic crisis period (Liagouras *et al.*, 2003; Livanos, 2010). Hence, the protective effect might exist only for those with extremely high education, which might work on their field of expertise and have better career prospects.

In general, the results of the present study have to be examined in parallel with the effects of the recent economic crisis on Greece. The unemployment and insecurity for younger people (Frangos *et al.*, 2012) could be responsible for their worse mental health compared to older participants. Similarly, the worse mental health of students could be explained by such an effect. Yet, this might not account only for Greece, since a wide range of studies across different populations confirms that students have high rates of mental health problems (Al-Daghri *et al.*, 2014; Auerbach *et al.*, 2016; Bayram and Bigel, 2008).

Finally, the higher levels of somatisation of the private sector workers compared to public sector workers and freelancers, is a quite interesting finding with no obvious explanation. It could be supported that public sector workers experience higher levels of security in their employment status (since according to Greek laws they can’t be fired), while freelancers experience a higher degree of freedom and opportunities for further financial growth. However, private sector workers do not experience any of those benefits. As supported by Ford (1997), somatisation is experienced because of inability to express the psychological burden, a justified and forced “inability” in a workplace environment. Thus, it might be easier for private sector workers to somatise their emotional burden.

A few limitations have to be reported concerning this study. Firstly, some sociodemographic data such as participants’ body mass index (BMI), the presence of chronic disease and the income status were not assessed. The study followed a convenient sampling approach, nevertheless the sample size was quite large and was recruited from two different areas of the country (urban and rural). Although mental health was assessed with self-reported instruments with high psychometric properties, the use of interviews is considered as a more reliable way to study depression (Robson, 2002).

Based on the findings and the limitations of the present study, some suggestions could be made for future research. As quantitative studies are not extremely reliable to shed light on the mechanism of the studied phenomena (Robson, 2002), the use of qualitative methods (*e.g.* interviews) could be more reasonable to investigate the potential mechanism for these effects (*e.g.* the worse mental health of smokers and young people), to provide in-depth explorations, and to form relevant theories (Babbie, 2013).

As for practical implications, the high percentage of participants experiencing moderate or severe distress (42.9%) indicates the need to develop stress-management interventions in the community. Finally, this study highlighted specific populations, such as students and those aged 18–34 years, who experience poor mental health and high somatisation. For that reason, public health policy makers should focus on the development of interventions aiming at the improvement of mental and physical well-being especially for those age groups.

## Figures and Tables

**Table 1.** Sociodemographic characteristics of the study participants.

Characteristic	Absolute value (%)
**Sex**	
Female	1.458 (60.1%)
Male	967 (39.9%)
**Age**	
18–34	l.268 (52.3%)
35–49	754 (31.3%)
50–64	369 (15.2%)
65 or more	34 (1.4%)
**Area of Residence**	
Athens	1.791 (73.9%)
Giannitsa	634 (26.1%)
**Family status**	
Married	834 (34.4%)
Unmarried, living alone	1.022 (42.1%)
Unmarried, living with a partner	417 (17.2%)
Widowed or divorced	151 (6.2%)
**Number of children**	
None	1.584 (65.3%)
One	385 (15.9%)
Two	374 (15.4%)
Three or more	82 (3.4%)
**Educational status**	
Primary	11 (0.5%)
Gymnasium	18 (1.6%)
Lyceum	661 (27.3%)
Vocational training	300 (12.4%)
Tertiary	1.033 (42.6%)
MSc	343 (14.1%)
PhD	39 (1.6%)
**Occupational status**	
Private sector worker	801 (33%)
Freelancer/businessman	560 (23.1%)
Public sector worker	474 (19.5%)
Student	396 (16.3%)
Unemployed	138 (5.7%)
Pensioner	38 (1.6%)
Houseworker	18 (0.7%)
**Smoking status**	
Non-smoker	1.387 (57.2%)
Current smoker	741 (30.6%)
Occasional smoker	297 (12.2%)

**Table 2.** Prevalence of levels depression, anxiety, distress and somatisation, according to the 4-DSQ.

Number of Participants (%)			
	Normal or mild	Moderate	Severe
**Depression**	1,803 (74.4%)	358 (14.8%)	262 (10.8%)
**Anxiety**	1,582 (65.3%)	549 (22.7%)	290 (12.0%)
**Distress**	1,381 (57.1%)	725 (30.0%)	314 (13.0%)
**Somatisation**	1,713 (70.8%)	577 (23.9%)	128 (5.3%)

**Table 3.** Prevalence of levels depression, anxiety, distress and somatisation, by sex.

Number of Participants (%)				
	Males		Females	
	Normal, mild or moderate	Severe	Normal, mild or moderate	Severe
**Depression**	883 (91.4%)	83 (8.6%)	1,324 (90.9%)	133 (9.1%)
**Anxiety**	904 (93.8%)	60 (6.2%)	1,322 (90.7%)	135 (9.3%)
**Distress**	877 (91.1%)	86 (8.9%)	1,229 (84.4%)	228 (15.6%)
**Somatisation**	946 (98.0%)	19 (2.0%)	1,368 (94.2%)	85 (5.8%)
**Total**	967 (100%)		1.458 (100%)	

**Table 4.** Associations of participants’ sociodemographic variables and the 4-DSQ sub-scales.

	Distress sub-scale		Anxiety sub-scale		Depression sub-scale		Somatisation sub-scale	
	Mean value (S.D)	P	Mean value (S.D)	P	Mean value (S.D)	P	Mean value (S.D)	P
**Sex**		**0.000**		**0.000**		**0.593**		**0.000**
Male	8.52 (7.22)		2.34 (3.84)		1.34 (2.64)		5.56 (5.22)	
Female	10.79 (7.99)		3.23 (4.31)		1.40 (2.67)		8.65 (6.28)	
**Age**		**0.000**		**0.000**		**0.009**		**0.325**
18–34	10.92 (7.89)		3.42 (4.36)		1.55 (2.78)		7.65 (6.04)	
35–49	9.05 (7.52)		2.38 (3.86)		1.18 (2.48)		7.24 (5.99)	
50–64	8.20 (7.40)		2.02 (3.72)		1.18 (2.54)		7.17 (6.35)	
65 or more	8.14 (7.07)		2.94 (4.53)		1.12 (3.05)		6.69 (0.12)	
**Area of residence**		**0.178**		**0.604**		**0.602**		**0.336**
Athens	10.01 (7.82)		2.85 (4.18)		1.39 (2.72)		7.36 (5.94)	
Giannitsa	9.53 (7.63)		2.95 (4.10)		1.33 (2.48)		7.63 (6.40)	
**Family status**		**0.000**		**0.000**		**0.000**		**0.005**
Married	8.34 (7.33)		2.12 (3.57)		1.05 (2.35)		7.15 (6.04)	
Unmarried, living with a partner	10.84 (8.27)		3.13 (4.16)		1.54 (2.84)		8.22 (6.09)	
Unmarried, living alone	10.72 (7.76		3.37 (4.44)		1.57 (2.79)		7.22 (5.95)	
Widowed or divorced	10.12 (7.52)		3.01 (4.53)		1.45 (2.77)		8.28 (6.71)	
**Number of children**		**0.000**		**0.000**		**0.001**		**0.955**
None	10.59 (7.86)		3.20 (4.30)		1.51 (2.75)		7.43 (5.94)	
One	8.66 (7.23)		2.39 (3.90)		1.01 (2.29)		7.57 (6.26)	
Two	8.16 (7.19)		2.05 (3.44)		1.10 (2.51)		7.33 (6.26)	
Three or more	9.90 (9.08)		2.74 (4.64)		1.80 (3.09)		7.32 (6.74)	
**Educational status**		**0.010**		**0.000**		**0.000**		**0.000**
Primary	7.63 (11.02)		3.18 (6.63)		1.54 (3.38)		8.90 (10.24)	
Gymnasium	11.26 (8.11)		4.10 (5.42)		2.44 (3.64)		8.28 (6.78)	
Lyceum	10.59 (8.13)		3.38 (4.54)		1.75 (3.02)		7.57 (6.44)	
Vocational training	9.52 (7.66)		2.96 (4.18)		1.12 (2.22)		8.35 (6.19)	
Tertiary	9.81 (7.65)		2.72 (3.93)		1.26 (2.54)		7.40 (5.88)	
MSc	9.34 (7.39)		2.29 (3.78)		1.17 (2.52)		6.72 (5.52)	
PhD	6.63 (6.66)		1.58 (2.63)		0.53 (1.46)		4.02 (4.18)	
**Occupational status**		**0.000**		**0.000**		**0.000**		**0.000**
Unemployed	12.12 (8.51)		4.25 (5.06)		2.22 (3.54)		8.10 (6.44)	
Student	11.98 (7.97)		4.17 (4.77)		1.82 (3.03)		7.82 (6.14)	
Public sector worker	8.63 (7.43)		2.26 (3.63)		1.04 (2.32)		6.85 (6.04)	
Private sector worker	9.78 (7.85)		2.85 (4.16)		1.34 (2.59)		8.10 (6.35)	
Freelancer/businessman	9.30 (7.22)		2.23 (3.56)		1.20 (2.42)		6.58 (5.34)	
Pensioner	7.34 (6.09)		1.89 (2.20)		0.94 (2.25)		6.64 (4.96)	
Houseworker	8.05 (9.82)		3.22 (6.19)		1.72 (3.30)		8.00 (6.07)	
**Smoking status**		**0.001**		**0.031**		**0.080**		**0.000**
Current smoker	10.51 (8.20)		3.07 (4.34)		1.55 (2.84)		8.25 (6.51)	
Non-smoker	9.37 (7.50)		2.69 (4.04)		1.28 (2.58)		6.97 (5.82)	
Occasional smoker	10.71 (7.73)		3.25 (4.19)		1.38 (2.58)		7.54 (5.86)	

## Key Points

- Approximately one third of an adult community sample in Greece suffered from moderate to severe mental health symptoms.
- Marriage, having children, non-smoking and higher educational status had protective effect on mental health.
- Students, unemployed and private sector workers had higher levels of depressive, anxiety, distress and somatisation symptoms.
- Youth’s mental health was worse than the elder’s.
- Sex differences were noted in all aspects of mental health symptoms except for depression.
